# Arthropod-Microbiota Integration: Its Importance for Ecosystem Conservation

**DOI:** 10.3389/fmicb.2021.702763

**Published:** 2021-08-02

**Authors:** Constanza Schapheer, Roseli Pellens, Rosa Scherson

**Affiliations:** ^1^Programa de Doctorado en Ciencias Silvoagropecuarias y Veterinarias, Campus Sur Universidad de Chile, Santiago, Chile; ^2^Laboratorio de Sistemática y Evolución, Departamento de Silvicultura y Conservación de la Naturaleza, Universidad de Chile, Santiago, Chile; ^3^UMR 7205, Muséum National d’Histoire Naturelle, Centre National de la Recherche Scientifique, Ecole Pratique de Hautes Etudes, Institut de Systématique, Évolution, Biodiversité, Sorbonne Université, Université des Antilles, Paris, France

**Keywords:** mutualism, planetary boundaries, ecosystem engineers, ecosystem services, detritivore arthropods

## Abstract

Recent reports indicate that the health of our planet is getting worse and that genuine transformative changes are pressing. So far, efforts to ameliorate Earth’s ecosystem crises have been insufficient, as these often depart from current knowledge of the underlying ecological processes. Nowadays, biodiversity loss and the alterations in biogeochemical cycles are reaching thresholds that put the survival of our species at risk. Biological interactions are fundamental for achieving biological conservation and restoration of ecological processes, especially those that contribute to nutrient cycles. Microorganism are recognized as key players in ecological interactions and nutrient cycling, both free-living and in symbiotic associations with multicellular organisms. This latter assemblage work as a functional ecological unit called “holobiont.” Here, we review the emergent ecosystem properties derived from holobionts, with special emphasis on detritivorous terrestrial arthropods and their symbiotic microorganisms. We revisit their relevance in the cycling of recalcitrant organic compounds (e.g., lignin and cellulose). Finally, based on the interconnection between biodiversity and nutrient cycling, we propose that a multicellular organism and its associates constitute an Ecosystem Holobiont (EH). This EH is the functional unit characterized by carrying out key ecosystem processes. We emphasize that in order to meet the challenge to restore the health of our planet it is critical to reduce anthropic pressures that may threaten not only individual entities (known as “bionts”) but also the stability of the associations that give rise to EH and their ecological functions.

## Introduction

Recent reports from the United Nations’ Intergovernmental Science-Policy Platform on Biodiversity and Ecosystem Services (IPBES), declared that the Earth’s ecosystem health has become progressively worse at an unprecedented accelerating pace [Intergovernmental Science-Policy Platform on Biodiversity and Ecosystem Services (IPBES) and United Nations, 2020]. Our species has put at risk much of Earth’s biodiversity and ecosystem processes (Brooks et al., 2002; Pievani, 2014; Lade et al., 2019). The loss of biodiversity and damage to ecosystems is of such a magnitude that it is being directly linked to current global crises (i.e., sanitary, developmental, economic, security, and social). The diverse initiatives and efforts to biodiversity decline, such as the “Sustainable Development Goals” (Glossary), have had no real impact on biodiversity conservation and even less on ecological process restoration (Zeng et al., 2020). It has been proposed that only true “transformative changes,” aimed not only at protecting, but also at restoring nature’s critical condition will change the current biodiversity and environmental health decline [Intergovernmental Science-Policy Platform on Biodiversity and Ecosystem Services (IPBES) and United Nations, 2020]. Coherently, the decline of “genetic biodiversity” (i.e., biodiversity loss; Hooper et al., 2012) and alterations of the biogeochemical cycles (Glossary; Sabater, 2008; Pinder et al., 2013; Nielsen and Ball, 2015) have been considered among the most severely compromised Planetary Boundaries (Glossary; Rockström et al., 2009; Steffen et al., 2015).

These two Planetary Boundaries, biodiversity and biogeochemical cycles are not independent. Ecosystem processes such as nutrient cycles are highly dependent on biological diversity (Lacroix and Abbadie, 1998; Falkowski et al., 2008; Gamfeldt et al., 2008; Naeem et al., 2012; Bishop et al., 2020). Even the faculty of ecosystems to sustain multiple and simultaneous functions and services (i.e., ecosystems’ multifunctionality properties, see Glossary) are only possible when a highly diverse community is considered (Hector and Bagchi, 2007; Maestre et al., 2012). This calls into question the idea of function redundancy with “expendable” elements in biodiversity (Hector and Bagchi, 2007; Allan et al., 2015) and challenges us to develop new perspectives to apply this knowledge to conservation and the restoration of the environment.

A canonical example of the importance of community diversity for maintaining ecosystem health is the role of its above-mentioned multifunctional properties in relation to planet Earth’s nutrient cycles (Glossary; Crawford, 2005). To understand this case, it is necessary to review the role of microorganism communities as the basic elements connecting ecosystem processes with biodiversity. The history of life on our planet and the constancy of its ecosystems have depended largely on microorganism community functional diversity, allowing the Earth to be habitable by other (uni- or multi-cellular) life forms (Margulis, 1993; Kasting and Siefert, 2002; Hohmann-Marriott and Blankenship, 2011). These biogeochemical processes are until today essential for the maintenance of life on earth (Madsen, 2011). Microbial metabolism and biogeochemical cycles developed together in such a way that they shaped our planet atmosphere (Falkowski et al., 2008; Fenchel et al., 2012). The first forms of microbial life, more than 3.7 million years ago, were exposed to an environment full of toxic gases such as dinitrogen (N_2_), carbon dioxide (CO_2_), methane (CH_4_), and ammonia (NH_3_), among others (Margulis, 1993; Falkowski et al., 2008; Barton and Northup, 2011; Dodd et al., 2017). Microorganism communities gradually adapted to these conditions, using these gases as energy sources (Margulis, 1993; Kasting and Siefert, 2002; Hohmann-Marriott and Blankenship, 2011). Microorganism biodiversity relevance as the foundation of ecosystem dynamics is apparent even today in the cycles of the six mayor elements; H, C, N, O, S, and P (Falkowski et al., 2008). Particularly soil nitrogen cycle depends on microorganism communities; bacteria, fungi, and archaea and their diversity have been found associated with N in all its phases; fixation, mineralization, nitrification and denitrification (Hayatsu et al., 2008). Nitrogen is key to life on our planet, since it constitutes a prime part of the molecules that make up living organisms such as DNA, RNA, amino acids and proteins. However, it is currently thought that anthropic effects such as intensive agriculture, livestock and indiscriminate use of fertilizers are causing deep alterations to this cycle (Guiry et al., 2018; Wang et al., 2018), which is one of the above-mentioned global planetary boundaries exceeded (Steffen et al., 2015). Considering the interconnectedness and relevance of biodiversity for the protection and restoration of ecosystem health, it is key to understand the anthropic effects on microbial communities (Falkowski et al., 2008; Gillings and Paulsen, 2014). Thanks to methodological and epistemological advances in the study of biological interactions, novel venues have opened allowing to ponder the importance of microorganism communities (Krumbein, 1996; Barton and Northup, 2011). Therefore, in this work we propose it is necessary to incorporate this body of knowledge to biodiversity conservation in order to a more effective contribution to the maintenance of ecological processes that allow the Earth ecosystems’ functioning.

Microorganisms’ lifestyles can be broadly classified as free-living or associated with a multicellular host (Barton and Northup, 2011). In this work we review the relevance of current knowledge regarding this second major group (i.e., associated with a multicellular host, in an exo or endosymbiotic association, see Glossary) for the preservation and restoration of threatened nature. Specifically, our objectives are: (1) To review the ecosystem importance of the symbiosis between microorganisms and terrestrial arthropods in key processes for the maintenance of ecosystems (i.e., biogeochemical cycles), (2) To discuss how anthropic pressures (pollution and loss of habitat) can alter this symbiosis, and (3) debate the possibilities and challenges of incorporating this conceptual framework into conservation sciences in the current global scenario.

## Multicellular Associations: the Ecosystem Holobiont

**FIGURE 1:**
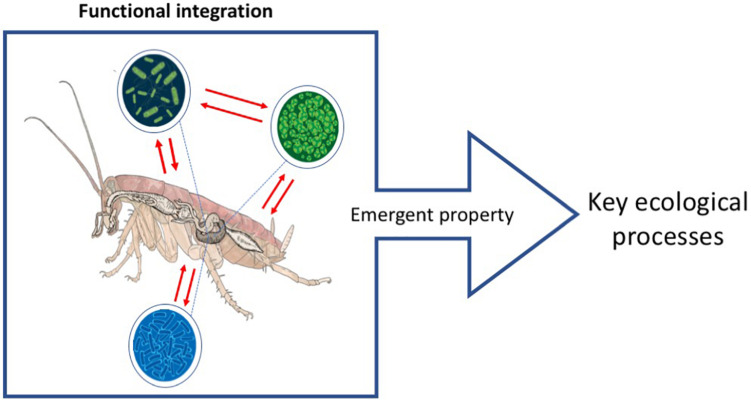
Ecosystem holobiont. The red lines show the functional integration between bionts resulting in the development of emergent properties with ecosystemic functions. For the case of the ecosystem holobiont, these properties are key ecosystem process such as the sustaining of nutrient cycling. Scheme based on the proposals of Catania et al. (2016) and Suárez (2020).

**FIGURE 2:**
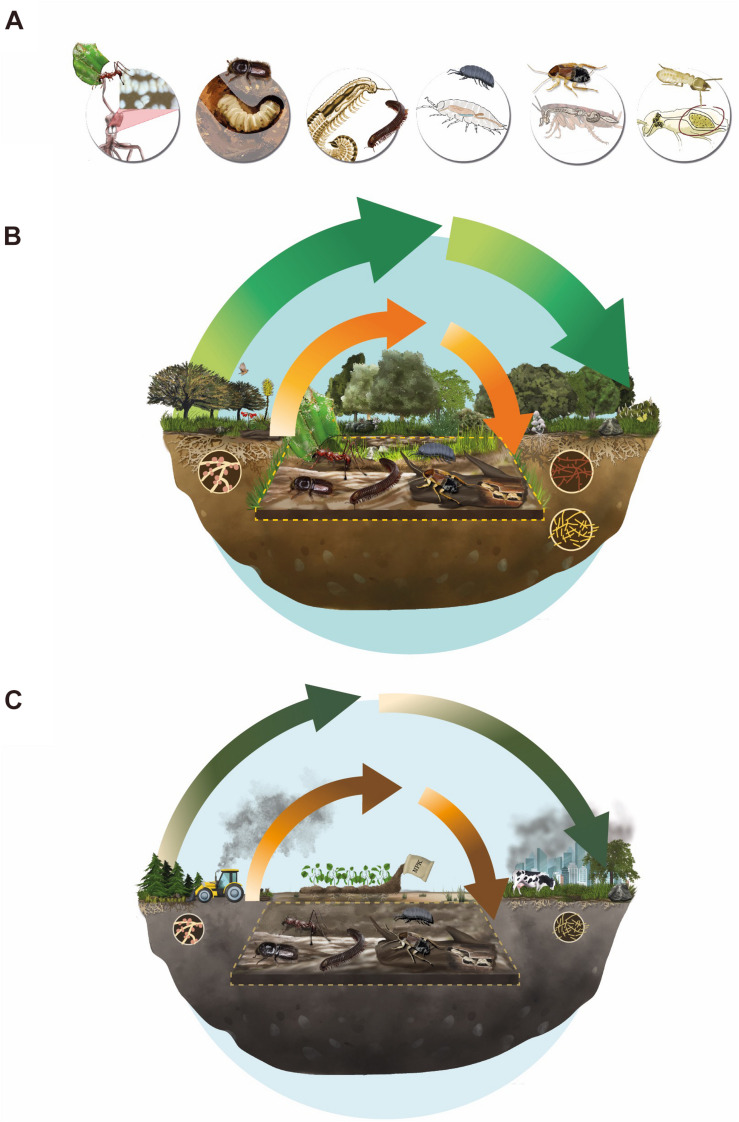
Emergent properties. **(A)** Examples of arthropods comprising Ecosystem Holobionts (EH) in conjunction with their associated microbiological bionts, from left to right: (i) ants, (ii) bark beetle, (iii) millipedes, (iv) woodlice, (v) cockroaches, and (vi) termites. These animal-microorganism associations are also represented in two contrasting scenarios: an undisturbed ecosystem **(B)** and a human-altered ecosystem **(C)**. For **(B,C)** green arrows show nutrient cycles produced by the action of free-living microorganisms, and orange arrows the cycles in which EHs participate (within the dotted line). Arrows width illustrates the degree of anthropogenic disturbances impacting nutrient cycling processes, with thinner lines indicating greater degree of alteration.

All multicellular organisms, e.g., animals and plants, have symbiotic interactions with microorganisms (Zilber-Rosenberg and Rosenberg, 2008; Bordenstein and Theis, 2015; Carthey et al., 2019). Microbiota and their hosts (either plants or animals) are capable of performing different ecosystem functions in a way that could not take place in the absence of these interactions (Koide, 2000; Selosse et al., 2004; Peirano, 2007; Tripp et al., 2017). In an evolutionary context, these phenomena have also been described as hologenomic adaptations (see Glossary), where new adaptive traits emerge as a result of the symbiosis between microorganisms and their multicellular hosts (Finlay et al., 1997; Kitade, 2004; Korb, 2007; Six, 2013; Dietrich et al., 2014; Zepeda Mendoza et al., 2018; Bredon et al., 2020; Suárez, 2020; Suárez and Triviño, 2020). These emergent properties are often associated to fundamental ecological processes that contribute to the maintenance of ecosystems, such as matter and energy flows (Figures 1, 2 and Supplementary Table 1). Ecologically sound examples are the dependence of plants on microorganisms in the rhizosphere (Mendes et al., 2013), the obligatory intracellular symbionts of vertebrates and invertebrates (Sabree et al., 2009; Tokuda et al., 2013; Ju et al., 2020) as well as macroalgae and their associated microbiota. In all these examples these associations’ emergent properties fulfill key ecosystem functions (van der Loos et al., 2019). Unfortunately, all of these processes are being strongly affected by anthropic activities (Marzinelli et al., 2018). These delicate key processes have been evidenced extensively in plants, for instance in the participation of plant microbiota in the biogeochemical cycles of nitrogen and methane, thanks to the interaction with microorganisms present in the rhizosphere (Turner et al., 2013). In this process human-derived perturbations have been found to arrest the contribution of this association in nutrient cycling (Turner et al., 2013). Likewise, the carbon and phosphorus cycles, fundamental in the ability of forests to store carbon dioxide, are carried out by trees in association with *Rhizobium* spp. (Batterman et al., 2013) and *Rhizobacteria* spp. (Granada et al., 2018).

Microbial associations have also been linked to the regulation of different ecological dynamics, for example the association between coral and dinoflagellate colonies, which form large reefs allowing the development of a miscellaneous community including fish and marine invertebrates, among others (Frade et al., 2008). There are also examples of the emergent benefits of microbial interactions with vertebrates, such as the case of carrion-feeding birds (Accipitriformes: Cathartidae). These have a strong adaptation to eat carrion thanks to the diversity of the microbial community of their skin. The processes of decomposition of organic matter of animal origin, and its further nutrient cycling, are accelerated through the action of these birds (Roggenbuck et al., 2014). Terrestrial invertebrates and their associated microorganisms have a fundamental role in the processes of plant-derived organic matter cycling and the subsequent nutrient flow (Hättenschwiler and Gasser, 2005; da Silva Correia et al., 2018). Such is the recurrence and value of these associations in nature that multicellular organisms, along with their community of microorganisms (microbiota), are currently being considered as an integrated and organized unit called “Holobiont” (see Glossary). This corresponds to a functional unit of life itself, capable of emergent properties that cannot be carried out by their parts or “bionts” (see Glossary) separately (Meyer-Abich, 1943; Margulis and Fester, 1991; Zilber-Rosenberg and Rosenberg, 2008; Guerrero et al., 2013; Six, 2013; Bordenstein and Theis, 2015; Theis et al., 2016; Carthey et al., 2019; Bredon et al., 2020). Although this definition has been criticized, mainly because it is still debated whether holobionts would constitute an evolutionary unit or unit of selection (Moran and Sloan, 2015; Douglas and Werren, 2016; Skillings, 2016; Stencel and Wloch-Salamon, 2018). Beyond that discussion, microbiota actively participating in biological processes such as reproduction and nutrition that have been described in all multicellular organisms (Moya and Ferrer, 2016; Gales et al., 2018; Ju et al., 2020; Supplementary Table 1). Alteration of the microorganism community leads to detrimental effects on their multicellular hosts in the vast majority of cases (Zhang et al., 2015; Warne et al., 2019).

However, beyond the evolutionary processes that underlie the existence of holobionts and further away, of understanding symbiosis as a mechanism of evolution (Klass et al., 2008; Zilber-Rosenberg and Rosenberg, 2008; Guerrero et al., 2013; Dietrich et al., 2014; Bordenstein and Theis, 2015; see symbiogenesis in Glossary), in this work we aim to incorporate and highlight the ecosystem aspects that make this integration a paramount phenomenon for the stability of key ecological processes. In addition of being relevant as selection units, holobionts are an ecosystemic unit with different elements integrated at a functional level (Catania et al., 2016; Doolittle and Booth, 2017; Lloyd and Wade, 2019; Suárez, 2020; Suárez and Stencel, 2020; Figure 1). This integrated unit is fundamental for ecosystems conservation as a whole. Thus, we propose the concept of “Ecosystem Holobiont” (hereafter “EH”) as a functional unit resulting from the integration of “bionts” composed by a multicellular organism together with its microorganism community, and the emergent properties that are generated as a result of the existence of this functional unit are indispensable for key ecological processes (Catania et al., 2016; Doolittle and Booth, 2017; Suárez, 2020; Suárez and Triviño, 2020; Glossary). EHs are fundamental for the adequate decomposition of recalcitrant organic compounds, e.g., lignin and cellulose, needed for nutrient cycling in soils (Guerrero et al., 2013; Six, 2013; Bredon et al., 2018, 2020; Supplementary Table 1).

In order to further illustrate the significance of the relation of EHs for biogeochemical cycles, we will review one milestone in Earth history that shows the relevance of microorganisms and multicellular integration in nutrient cycling. Between the Devonian and the Permian, 400 to 250 million years before present (MY), organic decomposition rates were increasing (Berner, 1990, 2001; Raymond et al., 2001). During the late Devonian, about 376–360 MY, the first trees appeared thanks to the development of evolutionary innovations such as lignin and cellulose (Algeo et al., 2001; Berner, 2001). These secondary plant compounds were difficult to digest for multicellular organisms that fed on plants living at that time (Martin, 1991; Calderón-Cortés et al., 2012). There is evidence of decomposition of wood material by lignolytic fungi from the late Carboniferous, 358.9–298.9 MY (Floudas et al., 2012). However, the activity of these fungi alone would not explain the increase in overall decomposition rates (Raymond et al., 2001). It has been then proposed that the radiation and evolution of detritivore invertebrates during the Carboniferous had a fundamental role in the decomposition of lignified plant litter (Nalepa et al., 2001; Kellogg and Taylor, 2004; Grimaldi and Engel, 2005). At first, detritivore invertebrates contributed by fractionating plant-derived materials mechanically, reducing them. At the same time, their foraging activity increased the contact surface with microorganism communities, facilitating decomposition (Shear and Kukalová-Peck, 1990; Crawford, 1992; Nalepa et al., 2001). This early exosymbiotic interaction (Glossary) between microorganisms and detritivores was only the beginning of an association that was slowly forged and cemented (Shear, 1991; Birkemoe et al., 2018). It has been suggested that this partnership was established through successive accidental intakes by invertebrates of microorganisms that were on the plant material (Ademolu et al., 2015). Then in the Mesozoic, 252 to 66 MY, different arthropod lineages, highly specialized in the decomposition of wood, developed complex interactions with a community of intestinal endosymbionts (see Glossary) and started to radiate (Grimaldi and Engel, 2005). There are fossil records of protists associated with cockroaches from the early Cretaceous (Poinar, 2009), as well as fossilized feces from these insects with leaves remnants, wood, cycad pollen and endosymbiotic protists (Hinkelman and Vršanská, 2020). The acquisition of flagellated symbionts during the late Jurassic period in lower termites allowed the hydrolysis of cellulose and fermentation products that were then absorbed by the host intestine (Bignell et al., 2010). Other forms of increasingly sophisticated symbiosis (Glossary) evolved by additional associations between arthropods and microorganisms, for example fungus-growing ants; where there is an intricate interaction between ants (Hymenoptera), fungi and actinomycete bacteria that probably originated during the Eocene (Schultz and Brady, 2008). Furthermore, today there are several cockroach lineages, such as Cryptocercidae, Blaberidae, Panesthinae and Zetoborinae (Blattodea), whose species show close intestinal endosymbionts interactions (Grandcolas and Deleporte, 1996; Guerrero et al., 2013) that allow this kind of EH to digest wood (Nalepa, 1984; Pellens et al., 2002; Brugerolle et al., 2003; Kitade, 2004; Korb, 2007; Bignell et al., 2010; Brune, 2014; Dietrich et al., 2014). Termites are also been considered “ecosystem engineers” (Glossary), since they play an irreplaceable role in nutrient cycling in arid and tropical ecosystems (Guerrero et al., 2013; Jouquet et al., 2016). For example, it has been found that termites contribute to soil physicochemical properties, soil turnover, water infiltration rate and soil microorganism diversity (Kaiser et al., 2017). However, this highly relevant function could not be carried out without the interaction between the multicellular (termite) organism and its complex intestinal endosymbiont assemblage (Kitade, 2004; Korb, 2007). The microorganism community associated with termites is composed of protists, bacteria and archaea (Brune, 2014). This EH organization is a necessary feature that confers the emergent properties that allow this entity to process lignified plant material, contributing to soil nutrient cycling as one of its emergent ecosystemic functions.

Considering these examples, it is also important to remind that the importance of the emergent properties resulting from the symbiosis between multicellular and microorganisms has been already emphasized by other authors (Catania et al., 2016; Suárez and Triviño, 2020). Therefore, there is both a corpus of evidences and rationale to contemplate the stability of the whole integration of bionts as the focus of conservation and restoration in order to preserve the associated processes they provide. A recent study developed in Burkina Faso illustrates the importance of EH; it showed that termite contribution to different aspects of ecosystem services depends on the species, and concomitantly on their obligatory association with intestinal endosymbionts (Kaiser et al., 2017). In the following section we develop further the relevance of terrestrial invertebrates along with their associated microorganisms and their ecosystem function.

## Terrestrial Arthropods as a Case of Ecosystem Holobionts

Along with the battery of digestive enzymes that arthropods produce endogenously (Watanabe and Tokuda, 2010), multicellular biont’s association with microorganisms allows this association to exploit low nutrient food resources (Six, 2013; Brune, 2014). In many cases the partnership with symbiotic microorganisms is essential for terrestrial arthropods to complete their life cycle (Kukor and Martin, 1983; Klepzig et al., 2009). Here we provide some examples of arthropods and their microbial associates, that could be considered EH under our proposal scope, focusing on their role in nutrient cycling in terrestrial ecosystems (Figures 1, 2A and Supplementary Table 1).

### Ants

These insects, belonging to the order Hymenoptera, family Formicidae (Ward, 2007), constitute the most abundant group of terrestrial arthropods, with an outstanding biomass of approximately 70 Mt of carbon (Tuma et al., 2020). To date, there are approximately 12,500 ant species described worldwide (Ward, 2010). Ants are widely recognized ecosystem engineers, mainly due to their influence on soil characteristics, nutrient cycling and resource availability (Jouquet et al., 2006; Tuma et al., 2020). For example, decomposition rates of cellulose-rich substrates have been shown to average 1.5 times higher and the net mineralization rates of N in *Pogonomyrmex rugosus* Emery, 1895 nests (Wagner and Jones, 2006). All these ecosystemic functions are carried out thanks to microbial partnerships. Ants have symbiotic relationships with multiple types of organisms, often involving more than two trophic levels (Ness et al., 2010), and are therefore considered multiparty symbiotic communities (Blatrix et al., 2009). Among the most remarkable and sophisticated examples are exosymbiotic relationships between leaf-cutter ants, fungi, yeasts and bacteria, where ants can set up farming systems thanks to these microorganisms (Schultz and Brady, 2008). These activities influence nutrient cycles by modifying primary productivity (Haines, 1975). It has been shown that N_2_ fixation exists in fungal gardens of at least eight species of leaf-cutter ants, as a result of nitrogen-fixing bacteria (Pinto-Tomás et al., 2009; Sapountzis et al., 2016). Two meta-analyses revealed that the refuse material from leaf-cutter ants nests show high levels of fertility, which constitute proper environments for plant growth (Farji-Brener and Werenkraut, 2015, 2017). Both the nests and the areas surrounding them, including refuse material areas, have a characteristic microbiota modulated by leaf-cutter ant activity; studies have suggested that these communities contribute to biodegradation and nutrient cycling processes (Lucas et al., 2017). Mound-building ants, from temperate and boreal forests, modify the properties of the soil where their mounds are located, generating soils with higher nitrogen and phosphorous content than the surrounding soil (Jurgensen et al., 2008). Studies have shown that the intestinal endosymbiont microbiota of leaf-cutter ants, which feed on fungal cultures, is composed of rhizobial N-fixing symbionts (Sapountzis et al., 2015). Ants have intestinal endosymbionts, for example intestinal bacterial communities have been found in *Cephalotes* spp. Latreille, 1802, that allow them to recycle nitrogenous residues from their diet (Hu et al., 2018). Diverse bacterial communities have been observed in the ileum of ant digestive system that may play a role in polysaccharide biodegradation (Bution and Caetano, 2008). This functional unit is strongly influencing nutrient cycling in terrestrial ecosystems; thus, it must be considered in the conservation actions that seek restoration. Once again while most efforts and resources in conservation policies are focused on charismatic vertebrates (Cardoso et al., 2011) relevant EH arthropods such as ants are being underestimated and unprotected. This must change in order to revert current global ecosystemic decline.

### Bark Beetles

These coleopterans and their microorganism partners stand out as a case in which the resulting EH from this association is extremely important for the ecosystemic role of biodegradation, i.e., decomposition of recalcitrant plant material, such as cellulose and lignin. Therefore, this EH is very important for carbon cycling in its environment. Bark beetles belong to the Curculionidae family (subfamilies Scolytinae and Platypodinae). There are currently around 3,500 species that build and inhabit galleries under the bark of trees where they spend most of their life. It is also where they lay eggs; the larvae feed and develop in the same substrate (Kirkendall et al., 2015). These coleopteran bionts have symbiotic interaction with a variety of filamentous fungi and yeasts, mostly Ascomycetes of the genera; *Ophthalmoma, Ceratocystiopsis, Grosmannia*, and *Ceratocystis* that are capable of degrading the xylem, which is then eaten by beetles (Breznak, 1982; Spatafora, 2001; Six and Wingfield, 2011). It has been discovered that essential nutrients for the development of beetles are available thanks to the action of these fungi associates (Ayres et al., 2000). Most bark beetle species carry spores, either over the exoskeleton cuticle or in specialized structures called mycangia (Six and Paine, 1999). It is possible to suggest these bionts may also help catalyzing the action of their microorganism partners by increasing their vagility through the ecosystem. Thus, these evidences support that this association can be considered as an EH. The impact of bark beetles on biogeochemical cycles (i.e., Carbon and Nitrogen) has been demonstrated in pine forests. It has been observed that the C/N ratio varied between pine plots with and without beetles, and was lower in the patches where they are found (Morehouse et al., 2008). This is significant also because it favors other detritivores to thrive, for instance woodlice. It has been proven that soils with low C/N ratio are more palatable (Gerlach et al., 2014) and maintain stable woodlouse populations (Kautz et al., 2000). In spruce forests (Pinaceae) that had been wiped out by bark beetles it was observed an increase in soluble N (NH_4_-N, organic N) and in P in the upper soil horizons, in contrast with plots without them (Kaňa et al., 2013). So far, most studies about bark-eating insects and their associated microorganisms have focused on the harm that these EHs do to trees: “the main model postulates that fungal associates of tree-killing bark beetles are responsible for overwhelming tree defenses and incurring in host tree mortality” (Six and Wingfield, 2011). However, it is necessary to rethink the importance of bark beetles, shifting from the perspective of a pest to be able to visualize their contribution in the cycling of nutrients. In this way we will be able to manage and conserve the processes that underlie their life cycle.

### Millipedes

Millipedes are arthropods of the Class Diplopoda. There are around 12,000 described species, but it is estimated that there could be around 80,000 and the vast majority are classified as detritivorous (Sierwald and Bond, 2007). They tend to be an important part of the litter arthropod biomass in different ecosystems (Crawford, 1976; Golovatch and Kime, 2009; Seeber et al., 2010). They are also essential in biodegradation of carbon and soil humification in terrestrial ecosystems (Alagesan, 2016; Pokhylenko et al., 2020; Supplementary Table 1). It has been found that the action of millipedes on leaf litter decreases the C/N ratio and improves some characteristics such as humidity and pH from acidic to neutral (Ashwini and Sridhar, 2006). There is also evidence that these invertebrates and their microbiota as a whole positively affect soil aggregation, levels of nitrogen and labile phosphorus (da Silva et al., 2017). The millipede gut is an ideal habitat for the development of symbiotic bacteria with enzymatic properties capable of cellulose and hemicellulose biodegradation (Taylor, 1982; Guzev and Byzov, 2006; Ramanathan and Alagesan, 2012). In the same fashion, yeast communities able to reuse uric acid have been found in millipedes’ hindgut, providing intestinal bacteria with compounds rich in nitrogen (Byzov et al., 1993). Millipedes can also influence the free microbial leaf litter community. It was observed that leaf litter fragmentation and the presence of the feces produced by this EH increased the CO_2_ release due to microbial metabolism, which has positive effects on decomposition rates (Suzuki et al., 2013). It has recently been observed that these arthropods are susceptible to urbanization, showing a reduction in species and functional richness in anthropized habitats (Tóth and Hornung, 2019).

### Woodlice

Another astounding example are the terrestrial isopods (Oniscidea), also known as woodlice. These terrestrial crustaceans are among the indispensable and most abundant detritivore soil macrofauna for many temperate habitats, actively participating in nutrient cycling (Hedde et al., 2007; Vos et al., 2011; Sutton, 2013; Zuo et al., 2014; Chen and Shaner, 2018; Pokhylenko et al., 2020). Woodlice also constitute a fundamental fraction of the macroarthropod fauna in tropical forests and nearby fast-growing plantations (Pellens and Garay, 1999). As with the case of termites, the importance of terrestrial isopods for ecological restoration has been highlighted due to their role in litter and soil nutrient cycling processes, accelerating the biodegradation of plant material (Snyder and Hendrix, 2008). For example, the enzymatic battery of the *Armadillidium vulgare* (Latreille, 1804), that breaks down polysaccharides (e.g., lignocellulose), is produced endogenously by this arthropod in association with its microbiota (Zimmer and Topp, 1998; Dittmer et al., 2016; Bredon et al., 2018). These enzymes have recently been described in detail for at least four woodlouse species, suggesting that this trait was one of the keys in the success of the colonization of terrestrial environments by this group (Bredon et al., 2020). Experiments have shown the positive impact of these arthropods on microbial respiration and macronutrient availability in the soil (Teuben, 1991; Kautz and Topp, 1999). Considering these findings, woodlouse conservation should be a priority to protect and maintain biogeochemical cycles. The significance of these arthropods as bioindicators of ecosystems health status has been already emphasized (Paoletti and Hassall, 1999; Solomou et al., 2019), together with their ability to accumulate heavy metals in their hepatopancreas (midgut caeca) vesicles (Hopkin and Martin, 1982; Papp et al., 2019). However, there is no certainty on the impact that this could have on their performance as detritivores, since this tissue also harbors microorganisms related with cellulose and lignin biodegradation (Hobbelen et al., 2004; Dittmer et al., 2016; Supplementary Table 1).

### Cockroaches

Cockroaches belong to the order Blattodea, and have more than 4,600 species described (Princis, 1962-1971; Beccaloni and Eggleton, 2013), which is likely to represent one fourth of their total diversity (Pellens and Grandcolas, 2007). Despite their bad reputation, only 20–30 (less than 1%) of these species are considered pests (Bell et al., 2007). The remaining cockroaches correspond to native species inhabiting diverse wild environments from deserts to tropical forests, where they are most diverse (e.g., 644 spp. in Brazil; 238 spp. in the Guiana Shield - Pellens and Grandcolas, 2007; Evangelista et al., 2017, respectively). Cockroaches can be found in a great diversity of microhabitats from the canopy to underground caves and tree holes, and often move across habitats in spite of habitat specialization (e.g., Grandcolas, 1993a; Weinstein, 1994; Pellens et al., 2007b, 2011). Some species are known to contribute to specific processes such as seed dispersal (Uehara and Sugiura, 2017), plant pollination (Nagamitsu and Inoue, 1997; Vlasáková, 2015; Suetsugu, 2019) and probably as scavengers in birds’ nests (Van Baaren et al., 2002). But their most generalized contribution is to the breakdown of organic debris (Nalepa et al., 2001; Sabo et al., 2005; Bell et al., 2007). One extreme specialization of cockroaches’ concerns wood-feeding, as it involves complex interactions with flagellates (Mastigophora) or ciliates (Ciliophora). This association conform an EH directly involved in the decomposition of cellulose and lignin (Grandcolas, 1995; Pellens et al., 2002; Brugerolle et al., 2003; Berlanga et al., 2016), and mechanisms that assure the transmission of endosymbionts among individuals and across generations. These interactions, related to life in a confined environment and to a nutrient-poor diet, have been hypothesized to be at the origin of sub-social behavior in Dictyoptera, which appeared independently in very different lineages (Pellens et al., 2007a; Klass et al., 2008; see also Grandcolas, 1993b and Murienne et al., 2008 for examples of xylophagy in other families). Although much remains to be studied about the role of cockroaches and their microorganism partners as EH involved in the decomposition of organic matter, available results suggest that their impact is not negligible. Thanks to the alliance between these insects their gut microbiota bionts, this EH is capable of digesting recalcitrant plant materials (Prins, 1991). When hosted in the cockroach gut, its symbiotic bacteria produce enzymes capable cellulose biodegradation, contributing with this emergent ecosystemic property (Cruden and Markovetz, 1987). Greater species richness was found in places in tropical forests with high phosphorus content, an interesting result since there may be a relationship between the action of cockroaches and the availability of this element (Tarli et al., 2014). Experimental studies have shown that the intestinal microbiota of different groups of cockroaches are usually quite stable, independent of their diet (Lampert et al., 2019). This presumably provides flexibility in the use of food resources, positioning these insects as an important element of soil trophic networks (Ardestani et al., 2020). Their ability to survive on diets poor in nitrogen thanks to the symbionts within their fat bodies (*Blattabacterium* spp.) allow them to develop in substrates with a high C/N ratio, which would put this cockroach- *Blattabacterium* interaction in the first line of recalcitrant organic compound biodegradation (Sabree et al., 2009; Tokuda et al., 2013). Although the ecosystem relevance of cockroaches and their associates have been recognized, the scientific literature regarding this group and its microbiota in wild environments is scarce, so the detritivore role of these EHs is still not fully understood. For example, cockroaches can harbor nitrogen-fixing bacteria in their gut microbiota (Cruden and Markovetz, 1987), therefore their participation in biogeochemical cycles is most likely being underestimated. Due to the fact that few research groups are currently studying this wild EH, i.e., most cockroach research is focused on pest species, their relevance has been largely underestimated. Although more research is needed to fully understand their contribution, it is desirable that these associations can be considered in further conservation plans involving the cycling of nutrients in diverse terrestrial ecosystems.

### Termites

Termites (Blattodea: epifamily Termitoidea) are xylophagous organisms by excellence. Their global dry biomass is estimated to be around 50 Mt of carbon, which represents one fourth of total arthropod biomass (Tuma et al., 2020). They are capable of degrading cellulose and lignin thanks to the mutualistic interaction with their intestinal microbiota. This is composed mainly of prokaryotic organisms such as bacteria and archaea, and eukaryotes such as protists (Engel and Moran, 2013; Ni and Tokuda, 2013; Brune, 2014). Flagellates (Mastigophora) are also capable of breaking down cellulose, while prokaryotes contribute in the fermentation of soluble metabolites resulting from this breaking down (Brune and Stingl, 2005). It has been demonstrated that the association of termites and microorganisms is responsible for approximately 10% of the mineralization and biodegradation of carbon from soil litter in forests in Thailand (Yamada et al., 2005). In semi-arid ecosystems, such as the Australian Mulga (*Acacia aneura* F. Muell. ex Benth. – Fabaceae) forests, the partnership between termites and microorganisms’ contributes as the most relevant detritivorous function. Their gallery construction in the first 20 cm of the soil contributes to the degradation of organic matter, hence to plant and soil water retention capacity (Whitford et al., 1992). In tropical savannas, where termites are very abundant, it has been proposed that an important part of the CH_4_ balance is due to invertebrate-microorganism partnerships, since their activity contributes to the cycling of 21% of the methane produced by the soil (Jamali et al., 2011). Furthermore, in temperate ecosystems such as the forests of the southeastern United States, termites of the genus *Reticulitermes* Holmgren, 1913 (Rhinotermitidae) exert relevant influence on nutrient cycles, particularly increasing C and Ca in the soil (Myer and Forschler, 2019). In addition to intestinal endosymbionts, termites can have exosymbiotic (see Glossary) interactions with fungi, as is the case of *Termitomyces* spp. Heim, 1942 (Lyophyllaceae), which allows the degradation of recalcitrant materials from litter and wood before the ingestion by termites (Rouland-Lefèvre et al., 2006), this strategy reduces the C/N ratio of the substrate (Higashi et al., 1992). Nevertheless, despite their widely recognized importance in terrestrial ecosystems (Johnson and Whitford, 1975; Freymann et al., 2008; Jouquet et al., 2011; Harit et al., 2017), insufficient emphasis has been placed on studies of termite-microorganisms EH conservation worldwide, except for a few examples (see Davies et al., 2010, 2020; LeClare et al., 2020). Present-day evidences on their fundamental contribution to the stability of terrestrial ecosystems and thus the maintenance of Earth’s functioning should encourage their explicit inclusion in conservation and restoration programs and policy-making.

## Threats to Ecological Processes Mediated by Ecosystem Holobionts

Several anthropic pressures are causing negative impacts on EHs and their associated ecological processes (Figures 2B,C). Here we will briefly mention those that are most relevant, according to the available scientific literature. These hazards can be broadly classified into two related categories: (i) Contamination and (ii) Land use change. Both alterations are considered direct drivers of biodiversity loss (IPBES, 2019). Below we review some of the most relevant factors reported in the literature.

### Contamination

The presence of arthropods such as isopods and millipedes has long been considered a bioindicator of soil health status. However, the physiological state of these organisms is rarely considered, much less alterations in their microbiota and how this affects their performance. Evidence accumulated through decades points to the impact of chemical products, e.g., agrochemicals, pharmaceutical products and industrial and urban-derived contamination (e.g., sewage sludge production) on soil arthropod populations (Fox, 1964; Andrés et al., 2011; González-Alcaraz et al., 2020). There is a myriad of effects of chemical products on these organisms, such as weight loss, avoidance behavior and stress protein (hsp70) expression (but see van Gestel and Loureiro, 2018) for a study in which no negative consequences were reported). Nonetheless, there is a lack of studies evaluating the long-term and direct impacts of human-produced chemical products on terrestrial detritivore performance and subsequently on nutrient cycling. The closest is a recent study of gene expression against nickel exposure in *Porcellionides pruinosus* (Brandt, 1833) (Isopoda: Porcellionidae), showing a negative impact on oxidative stress, neurotoxicity and reproduction (Ferreira et al., 2019). Despite being a relevant contribution that explores the mechanisms behind the contamination effects on a soil arthropod, the microbiota dimension was not considered. Current evidence shows that antifungal and antibiotic chemical compounds released to the environment are affecting detritivorous invertebrates. For example, in livestock production, where antibiotic use is a common practice, there is evidence that it has negative consequences on dung beetle (Hammer et al., 2016) and springtail microbiota (Zhang et al., 2019), which can affect their performance and subsequently the nutrient cycles associated with their action. However, the mechanisms underlying this effect are still unclear (Lucas et al., 2019). Intensive agricultural management such as liming and fertilization can affect the intestinal microbiota of the edaphic fauna (Ding et al., 2019a,b). Recent work on soil collembolas showed that contamination with microplastics altered their gut microbiota and inhibited their growth (Zhu et al., 2018a). Other work showed that neonicotinoid and pyrethroid insecticides, widely used in agriculture, reduce the detritivore arthropods density (collembola, acari and diplopoda) as well as the rate of leaf litter decomposition (Pearsons and Tooker, 2021).

### Land Use Change

Habitat loss and degradation due to land use change are some of the main threats to biodiversity (Caniani et al., 2016); their impact on biological communities is evident both compositionally and functionally (Allan et al., 2015). These ecosystem modifications often alter the interactions of the microbiota. There have been successful initiatives regarding agricultural management in relation to the activity of detritivorous arthropods such as “set-aside,” which consists of farmers leaving part of their land out of intensive production. Although this type of management has been efficient in allowing the establishment of millipedes and woodlice (Tóth et al., 2016), it must be accompanied by a production system change that tends toward agroecology in order to reduce exogenous input such as the use of nitrogenous fertilizers and pesticides that alter nutrient cycles (Vitousek et al., 1997; Galloway et al., 2008) and disrupt arthropod populations (Sánchez-Bayo, 2011; Sánchez-Bayo and Wyckhuys, 2019).

The presence of antibiotic resistance genes has been proven in the collembolan microbiome. This phenomenon is related to different uses of soil by this arthropod (arable and park) (Zhu et al., 2018b). Land use changes can influence the increase of EH populations such as forest plantations and bark beetles and their associated microbiome, which could also alter nutrient cycles (Kaňa et al., 2013).

Tolerance to human alterations has been reported for mound-building termites in savannah ecosystems, since these insect’s nests persist despite advance of human alterations. Yet, the same authors point out the need to understand how human activities impacts the processes carried out by this EH (Davies et al., 2020). In another savannah study, it is shown that at small spatial scales (1–2 km patch diameter) natural-agricultural landscape heterogeneity strongly promotes termite foraging activity. Nonetheless, the opposite effect is seen at a larger landscape scale (5 km), concluding that landscape heterogeneity can disrupt nutrient cycling functioning of termite-microorganism associations (LeClare et al., 2020). Therefore these dynamics are still in need of further research. For instance, it is also necessary to consider the type of perturbation and the specific attributes of this EH. A study comparing primary forest, grazing areas and agricultural fields found greater termite richness and abundance in primary forests and grazing areas than in agricultural fields, and the structure of termite feeding groups differed significantly among the three types of land use (Muvengwi et al., 2017).

## Future Directions

Considering the conceptual framework proposed by IPBES for nature’s contribution to people (NCP) (see Glossary) the EH participates directly in at least two of them; NCP 1: habitat creation and maintenance; and NCP 8: formation, protection and decontamination of soils and sediments (Brauman et al., 2020) mainly through its participation in nutrient cycling and decomposition of recalcitrant materials (Supplementary Table 1). This means that this functional unit must be considered as an object of conservation.

We are facing difficult times when it comes to environmental conservation, but these are also times of great scientific discoveries; in recent decades we have seen a technological revolution that has allowed us a deeper understanding of the way in which organisms inhabit our planet (Madigan et al., 2015). Now we must take a further step toward understanding the biological interactions that make life on earth possible in order to restore and conserve them.

It is possible that the previous efforts to ameliorate Earth’s ecosystem crises have not been sufficient because canonically considered ecological included in these strategies, may not considered all key components underlying biodiversity’s ecosystem functions and their interrelationships. In order to develop strategies to cope with planetary boundaries such as biodiversity decline and biogeochemical cycles alterations cannot be regarded without considering EHs, because ecosystem processes such as nutrient cycles are highly dependent on biological diversity and its intricated interactions.

Conservation sciences must be nourished by updated methodological advances as well as epistemological discussions that seek to explain and understand the organization of life. In this way, the perspective of the EH’s functional integration provides us with a more complete view of nature as well as novel heuristic tools to face this challenge (Catania et al., 2016; Dupré, 2017; Suárez, 2020). Therefore, its incorporation into conservation policies and plans would provide concepts and methods to restore and preserve key processes (i.e., biogeochemical cycles) to guarantee ecosystems functioning in order to take actions that allow the restoration of key ecological processes for human survival.

## Concluding Remarks

The scientific community has recently provided evidence indicating that arthropod populations are declining in many places in the world, and that the leading causes of this decline are of anthropic origin (Hallmann et al., 2017; Lister and Garcia, 2018; Sánchez-Bayo and Wyckhuys, 2019; Eggleton, 2020; Wagner, 2020). As shown in this essay, this biodiversity loss may most likely also involve the decline of symbiotic microorganism diversity associated to these arthropods, which often result of emergent hologenomic adaptations (see Glossary). This involves a myriad of mechanisms to transfer this association between individuals from one generation to another. It is becoming well established that the loss of all these partnership of organisms drives to the loss of the associated ecosystem functions, leading to further alterations in biogeochemical cycles (Eggleton, 2020). At present day, the nitrogen and phosphorus cycles are already altered beyond acceptable limits to preserve human well-being and ecosystem resilience (Steffen et al., 2015). We propose that the loss of EHs (Figure 1 and Supplementary Table 1) might lead to severe alterations in ecosystem functioning (Figure 2), because it involves the depletion of entire microorganisms’ communities integrated with multicellular units and their associated key ecosystem functions. Therefore, the focus of conservation and restoration must take into consideration the integrity of these functional units in order to preserve the emergent processes they carry out (Figure 2). Without these EHs, it will be impossible to ensure that safe continuity of nutrient cycles. Specifically, the probability that nitrogen and phosphorus cycles remain within boundaries that do not threaten the safe operating space for humanity may be under threat by the loss of EHs.

Detritivore arthropods and their microorganism partners constitute an important portion of the invertebrate-microbial biomass in multiple ecosystems and have a fundamental role as EHs in Earth’s nutrient cycling (Supplementary Table 1). Therefore, it is key to consider them in conservation and restoration efforts of ecological processes (Paoletti et al., 2007).

In order to synthesize our proposal, in this study we did not delve into other arthropods that could be considered as EHs, such as Siricidae wasps, oribatid mites, dung beetles, saproxylic beetles or springtails, among many others (Supplementary Table 1). For example, *Sirex noctilio* Fabricius, 1793 (Hymenoptera: Siricidae), inoculate the fungus *Amylostereum areolatum* (Amylostereaceae) in the wood, using it as an external digester of lignocellulose compounds (Breznak, 1982; Thompson et al., 2014) and bacterial symbiosis (Adams et al., 2011). It was discovered that *Sirex cyaneus* also has active enzymes of fungal origin in its guts (Kukor and Martin, 1983). However, to the best of our knowledge the role of Siricidae wasps and their influence on nutrient cycling has not been studied yet, therefore we have not treated this case in depth (see Supplementary Table 1). The same applies to the influences of bark beetle EHs. Although their contribution to nutrient cycles has been demonstrated (Kaňa et al., 2013), it is necessary to comprehend their effect on the ecosystems beyond the paradigms of forestry production (Six and Wingfield, 2011). Apart from the fact that these EHs can provoke a decrease in forest production, the increase in their density due to forest management can have an impact on biogeochemical cycles. Finally, it is necessary to consider the microbiota associated with arthropods as part of a joint epigenetic inheritance system (Villagra and Frías-Lasserre, 2020), which may allow the maintenance of lineages and the attributes associated with EHs. Finally, It is also necessary to explore into evolutionary processes such as horizontal transfer of genes from microorganisms to arthropods (Faddeeva-Vakhrusheva et al., 2017; Bredon et al., 2019), in order to understand the importance of this integration and thus make efforts that allow its conservation in order to secure as well as restore ecosystem functioning on our planet.

## Author Contributions

All authors listed have made a substantial, direct and intellectual contribution to the work, and approved it for publication.

## Conflict of Interest

The authors declare that the research was conducted in the absence of any commercial or financial relationships that could be construed as a potential conflict of interest.

## Publisher’s Note

All claims expressed in this article are solely those of the authors and do not necessarily represent those of their affiliated organizations, or those of the publisher, the editors and the reviewers. Any product that may be evaluated in this article, or claim that may be made by its manufacturer, is not guaranteed or endorsed by the publisher.

## Glossary

**Biogeochemical cycles:** Cyclic flow of the biosphere elements, through a series of biochemical and geological processes (Jacobson et al., 2000).

**Bionts:** Organisms that make up the integration known as holobiont (Margulis and Fester, 1991).

**Ecosystem engineers:** Organisms whose actions modulate the availability of resources for other species (Jones et al., 1994).

**Ecosystem Holobiont:** Multicellular host and their associated microbiota participating in key ecological processes for the functioning of its ecosystems thanks to its holobiont-derived emergent properties (Figure 1, concept developed in this work).

**Ecosystem multifunctionality:** The ability of ecosystems to sustain multiple functions and services at the same time. This attribute is highly dependent on the biodiversity present in each ecosystem (Gamfeldt et al., 2008).

**Endosymbiont:** Symbiotic association where one of the associated organisms (bionts) inhabits inside the body of another biont (operational definition).

**Exosymbiont or ectosymbiont:** Symbiotic association where bionts inhabit different bodies (operational definition).

**Holobiont:** Functional symbiotic unit composed by the integration of multicellular and unicellular organisms. This holobiont occasionally generates a new features or attributes such as new morphological, physiological or immunological traits, among others. The holobiont concept has converged independently at different times possibly in the late 19th and early 20th century debates on the definition of symbiosis (Meyer-Abich, 1943; Margulis, 1990; Suárez, 2018; Baedke et al., 2020).

**Hologenome:** This correspond to multicellular host genome plus microbiome (Rosenberg et al., 2007; Zilber-Rosenberg and Rosenberg, 2008).

**Hologenomic adaptation:** Biological adaptations that have evolved in the holobiont or in a multispecies system (Suárez and Triviño, 2020).

**Microbiome:** Microbiota’s genome (Madigan et al., 2015).

**Microbiota:** Community of microorganisms that is normally located in multicellular host body (Madigan et al., 2015).

**Multicellular/Multicellular organisms:** Biont formed by more than one cell (Kaiser, 2001).

**Nature’s contribution to people:** Conceptual framework proposed by the Intergovernmental Science-Policy Platform on Biodiversity and Ecosystem Services (IPBES). This is based on the idea of ecosystem services (i.e., irreplaceable benefits for humanity provided by nature), but considering the interrelationship between humans and their ecosystems, and recognizing the role of local and indigenous people culture knowledge (Díaz et al., 2018).

**Planetary Boundaries:** Approach to global sustainability where safe operational limits for our species were defined. These limits include attributes of biodiversity, land use and biogeochemical cycles, among others (Rockström et al., 2009; Steffen et al., 2015).

**Sustainable Development Goals (SDGs):** Set of objectives developed at the summit of the United Nations Rio+20 in Brazil, 2012. These goals were proposed to reconcile environmental protection and socioeconomic development (Zeng et al., 2020).

**Symbiogenesis:** Mechanism by which evolution occurs as a result of symbiosis (Merezhkowsky, 1905; Sapp, 1994).

**Symbiosis:** In its most classical definition this correspond to biological interactions in which two entities differently named (different species) live together. Under this concept, cases of mutualism, commensalism and parasitism would be included under symbiosis conceptual umbrella (De Bary, 1879; Sapp, 1994; Oulhen et al., 2016; Suárez, 2018).
