# Supplementary material for: Arthropod-Microbiota Integration: Its Importance for Ecosystem Conservation
Source: Front Microbiol. 2021 Aug 2;12:702763. doi: 10.3389/fmicb.2021.702763 (PMC8365148; doi:10.3389/fmicb.2021.702763)
Supplement: Supplementary file 1 [file Table_1.docx]

| **Bionts** | | | | | **Type of association** | **Processes** | **References** |
| --- | --- | --- | --- | --- | --- | --- | --- |
| **Multiicellular organism** | | | **Microbiota** | |  |  |  |
| Malacostraca | Isopoda | *Ligia pallasii* (Ligiidae) | Bac | NA | EN: hepatopancreas | OMD: cellulose | Zimmer *et al*., 2002 |
|  |  | *Armadillidium vulgare* (Armadillidiidae) | Arc | Thaumarchaeota | EN: whole body | OMD: cellulose | Bredon *et al.,* 2018 |
|  |  |  | Bac | Proteobacteria, Actinobacteria Bacteroidetes, Firmicutes |  |  |  |
|  |  | *Porcellio scaber* (Porcellionidae) | Bac | Actinomycetes | EN: hepatopancreas | OMD: cellulose | Zimmer & Topp, 1998 |
|  |  |  | Fun | NA |  |  |  |
| Diplopoda | Spirostreptida | *Orthoporus ornatus (Spirostreptidae)* | Bac | Aerobic bacteria | EN: digestive tract | OMD: cellulose, hemicellulose and pectin | Taylor, 1982 |
|  | Spirobolida | *Comanchelus* sp. (Atopetholidae) |  |  |  |  |  |
|  | Sphaerotheriida | *Arthrosphaera magna* (Arthrosphaeridae) | Bac | *Alcaligenes faecalis, Citrobacter freundii, Bacillus pumilis, Corynebacterium* sp*, C. kutcheri, Pseudomonas aeroginosa, Streptococcus mitis, Lactobacillus fermentum, Micrococcus roseus, M. varians, Corynebacterium xerosis, Lacobacillus casei, Enterobacter aerogenes, Bacillus cereus* and *B. subtilis* | EN: digestive tract | OMD: starch, chitin, cellulose, proteins and hemicellulose | Ramanathan & Alagesan, 2012 |
|  | Spirobolida | *Aulacobolus newtoni* (Pachybolidae) |  |  |  |  |  |
|  | Spirostreptida | Spinotarsus colosseus (Odontopygidae) |  |  |  |  |  |
| Entognatha | Collembola | *Folsomia candida* (Isotomidae) | Bac | Proteobacteria, Actinobacteria, Bacteroidetes and Firmicutes | EN: digestive tract | OMD: recalcitrant polysaccharides | Agamennone *et al.,* 2019 |
| Insecta | Blattodea | *Reticulitermes flavipes* (Rhinotermitidae) | Bac | Acetogenic bacteria | EN: digestive tract | OMD: cellulose and hemicelluloses | Odelson & Breznak, 1983. |
|  |  | *Syntermes wheeleri* (Termitidae) | Arc | Methanobacteriales, Methanosarcinales, Methanomicrobiales and Euryarchaeota | EN: digestive tract | OMD: cellulose and hemicelluloses | Santana *et al.*, 2015 |
|  |  |  | Bac | Firmicutes |  |  |  |
|  |  |  | Fun | Dothideomycetes |  |  |  |
|  |  | *Globitermes brachycerastes* (Termitidae) | Bac | Spirochaetes, Firmicutes, Fibrobacteres, Bacteroidetes, Proteobacteria, Chlorobi, Synergistetes and Acidobacteria | EN: digestive tract | OMD: cellulose, hemicellulose and pectin | Liu *et al*., 2019 |
|  |  | *Nasutitermes corniger* (Termitidae) | Bac | Spirochaetes, Fimirmicutes, Bacteroidetes, Proteobacteria, Actinobacteria and Fibrobacteres | EN: digestive tract | OMD: cellulose and hemicelluloses | Köhler *et al*., 2012 |
|  |  | *Odontotermes yunnanensis* (Termitidae) | Bac | Bacteroidetes, Firmicutes, Proteobacteria, Spirochaetes, Synergistetes and Planctomycetes | EN: digestive tract | OMD: lignocellulose | Liu *et al*., 2013 |
|  |  | *Macrotermes* sp. (Termitidae) | Fun | *Termitomyces* spp. | EX: nest | NC: nitrogen fixation | Vesala *et al.*, 2019 |
|  |  | *Reticulitermes flavipes*  (Rhinotermitidae) | Bac | *Burkholderia* sp, *Dyella* sp., *Citrobacter* sp. and *Fontibacillus* sp. | EN: digestive tract | OMD: cellulose | Xie *et al.*, 2017 |
|  |  | *Macrotermes natalensis* (Termitidae) | Bac | Diazotrophic bacteria | EN: digestive tract | NC: nitrogen fixation | Sapountzis *et al.,* 2015 |
|  |  | *Odontotermes badius* (Termitidae) |  |  |  |  |  |
|  |  | *Macrotermes subhyalinus* (Termitidae) | Bac | Soil Bacteria | EX: termite runways (soil sheeting) | NC: nitrogen cycling | Ndiaye et al., 2004 |
|  |  | *Odontotermes nilensis* (Termitidae) |  |  |  |  |  |
|  |  | *Macrotermes muelleri* (Termitidae) | Bac | Ammonia-oxidizers and denitrifiers | EN: digestive tract | NC: ammonia oxidation and denitrification | Brauman *et al.,* 2015 |
|  |  | *Pseudacanthotermes militaris* (Termitidae) |  |  |  |  |  |
|  |  | *P. spiniger* (Termitidae) |  |  |  |  |  |
|  |  | *Coarctotermes clepsydra* (Termitidae) |  |  |  |  |  |
|  |  | *Capritermes capricornis* (Termitidae) |  |  |  |  |  |
|  |  | *Trinervitermes* spp. (Termitidae) |  |  |  |  |  |
|  |  | *Crenetermes albotarsalis* (Termitidae) |  |  |  |  |  |
|  |  | *Cubitermes* spp. (Termitidae) |  |  |  |  |  |
|  |  | *Thoracotermes macrothorax* (Termitidae) |  |  |  |  |  |
|  |  | Microcerotermes parvus (Termitidae) |  |  |  |  |  |
|  |  | *Nasutitermes nigriceps* (Termitidae) |  |  |  |  |  |
|  |  | *N. voeltzkowi* (Termitidae) |  |  |  |  |  |
|  |  | *Hodotermes mossambicus* (Hodotermitidae) |  |  |  |  |  |
|  |  | *Cavitermes tuberosus* (Termitidae) | Bac | Burkholderiales, Frankiales and Rhizobiales | EN: digestive tract / EX: nest | NC: nitrogen fixation | Hellemans *et al.,* 2019 |
|  |  | *Diploptera punctata* (Blaberidae) | Bac | Xanthomonadaceae, Sphingobacteriaceae, Lactobacillaceae, Caulobacteraceae, Rhizobiaceae and Methylophilaceae | EN: digestive tract | OMD: cellulose | Ayayee *et al.*, 2020 |
|  |  | *Parasphaeria boleiriana*  (Blaberidae) | Fla | Trichomonadida | EN: digestive tract | OMD: cellulose | Pellens *et al*., 2002 |
|  |  | *Blattella germanica* (Ectobiidae) | Bac | Bacteroidetes, Firmicutes, Proteobacteria and *Blattabacterium* | EN: adult and nymph alimentary tracts and fat bodies, embryos and ootheca | OMD: polysaccharides / NC: Nitrogen mobilization | Carrasco *et al.*, 2014 |
|  | Hemiptera | *Dactylopius coccus*  (Dactylopiidae) | Bac | *Dactylopiibacterium carminicum* | EN: ovaries | NC: nitrogen fixation | Vera-Ponce de León *et al*., 2017 |
|  |  | *D. opuntiae* (Dactylopiidae) |  |  |  |  |  |
|  | Coleoptera | *Euoniticellus intermedius* (Scarabaeidae) | Bac | Porphyromonadaceae, Comamonadaceae, Rhodobacteraceae and Sphingobacteriaceae | EN: digestive tract | OMD: cellulose / NC: nitrogen cycling | Shukla *et al.*, 2018 |
|  |  | *E. triangulatus (Scarabaeidae)* |  |  |  |  |  |
|  |  | *Onthophagus Ienzii* (Scarabaeidae) | Bac | Ammonifier, Actinomycetes | Exosymbiont: dung ball | NC: ammonification and nitrification | Kazuhira *et al*., 1991a; Kazuhira *et al*., 1991b |
|  |  |  | Fun | NA |  |  |  |
|  |  | *Melolontha hippocastani*  (Scarabaeidae) | Bac | Lachnospiraceae and Enterobacteriaceae | EN: digestive tract | OMD: hemicellulose / NC: Nitrogen cycling | Alonso-Pernas *et al*., 2016 |
|  |  | *Allomyrina dichotoma* (Scarabeidae) | Yea | *Spathaspora allomyrinae* | EN: digestive tract | OMD: fermentation of D-xylose | Wang *et al.*, 2016 |
|  |  | *Passalus* spp. (Passalidae) | Yea | *Lodderomyces, Scheffersomyces, Spathaspora, Sugiyamaella sp., Phaffomyces, Spencermartinsiella, Cryptococcus and Trichosporon* | EN: digestive tract | OMD: fermentation of cellobiose and xylose | Urbina *et al.*, 2013 |
|  |  | *Arrox agassizi* (Passalidae) |  |  |  |  |  |
|  |  | *Chondrocephalus* spp. (Passalidae) |  |  |  |  |  |
|  |  | *Ogyges* spp. (Passalidae) |  |  |  |  |  |
|  |  | *Oileus sargi* (Passalidae) |  |  |  |  |  |
|  |  | *Popilius eclipticus* (Passalidae) |  |  |  |  |  |
|  |  | *Proculus mnizechii* (Passalidae) |  |  |  |  |  |
|  |  | *Vindex* sp (Passalidae) |  |  |  |  |  |
|  |  | *Xylopassaloides chortii* (Passalidae) |  |  |  |  |  |
|  |  | *Veturius transversus* (Passalidae) | Yea | *Candida, Williopsis* and *Geotrichum* | EN: digestive tract | OMD: fermentation of D-xylose | Matos *et al*., 2017 |
|  |  | *Anoplophora glabripennis* (Cerambycidae) | Bac | NA | EN: digestive tract | NC: nitrogen fixation and recycling | Ayayee *et al*., 2014 |
|  |  | *Cyrtotrachelus buqueti* (Curculionidae) | Bac | *Lactococcus, Serratia, Dysgonomonas* and *Enterococcus* | EN: digestive tract | OMD: lignocellulose | Luo *et al.,* 2019 |
|  |  | *Dorcus rectus* (Lucanidae) | Yea | NA | EN: mycangia | OMD: xylose-fermenting | Tanahashi *et al.*, 2010 |
|  |  | *D. striatipennis* (Lucanidae) |  |  |  |  |  |
|  |  | *D. titanus sakishimanus* (Lucanidae) |  |  |  |  |  |
|  |  | *Prosopocoilus pseudodissimilis* (Lucanidae) |  |  |  |  |  |
|  |  | *Prismognathus angularis* (Lucanidae) |  |  |  |  |  |
|  |  | *Dendroctonus valens* (Curculionidae) | Bac | *Lactococcus, Acinetobacter, Pantoea, Rahnella, Stenothrophomonas, Erwinia, Enterobacter, Serratia, Janibacter, Leifsonia, Cellulomonas* and *Cellulosimicrobium* | EN: digestive tract | OMD: cellulose / NC: Nitrogen fixation | Morales-Jiménez *et al.,* 2009 |
|  |  | *D. rhizophagus* (Curculionidae) | Bac | *Raoultella terrigena* | EN: digestive tract | NC: Nitrogen fixation | Morales-Jiménez *et al.,* 2013. |
|  |  | *D. valens* (Curculionidae) |  |  |  |  |  |
|  |  | *D. terebrans* (Curculionidae) | Bac | *Enterobacter agglomerans* and *Enterobacter* spp. | N/N | NC: Nitrogen fixation | Bridges, 1981 |
|  |  | *Ips avulsus* (Curculionidae) | Bac |  |  |  |  |
|  | Hymenoptera | *Azteca trigona* (Formicidae) | Bac | *Bacillus* spp., *Pseudomonas* spp., *Enterobacter* spp. and Rhizobiales | EX: nest refuse | NC: nitrogen cycling | Lucas *et al.*, 2017 |
|  |  | Fungus-growing ants (Attine) | Bac | Mollicutes | EN: alimentary tracts | NC: nitrogen cycling | Sapountzis *et al*., 2019 |
|  |  | *Trachymyrmex cornetzi* (Formicidae) | Bac | Rhizobiales | EN: alimentary tracts | NC: nitrogen fixation | Sapountzis *et al*., 2018 |
|  |  | *Atta* spp. (Formicidae) | Bac | Enterobacteriaceae | EX: fungus gardens | OMD: lignocellulose | Aylward *et al.*, 2012 |
|  |  | *Acromyrmex* spp. (Formicidae) | Bac | Rhizobiales | EN: alimentary tracts | NC: nitrogen fixation | Sapountzis *et al*., 2015 |
|  |  | *Liometopum apiculatum* (Formicidae) | Bac | Firmicutes, Proteobacteria and Actinobacteria. | EN: alimentary tracts | OMD: starch, cellulose, hemicelluloses and lignin | González-Escobar *et al*., 2020 |
|  |  | Atta spp. (Formicidae) | Bac | *Pantoea* sp, *Klebsiella* sp. and *Azospirillum* sp. | EX: ant nests (fungal gardens) | NC: nitrogen fixation | Pinto-Tomás *et al.*, 2009 |
|  |  | Acromyrmex spp. (Formicidae) |  |  |  |  |  |
|  |  | *Sirex noctilio* (Siricidae) | Fun | *Amylostereum areolatum* | EN: mycangia | OMD: cellulose | Thompson *et al.*, 2013 |
|  |  | *S. noctilio* (Siricidae) | Bac | *Pantoea* spp. and *Streptomyces* spp. | EN: mycangia | OMD: cellulose | Adams *et al*., 2011; Takasuka *et al*., 2013 |

**Table 1:** Summary of ecosystem processes mediated by the integration of arthropod and microorganism taxa bionts conforming the Ecosystem Holobiont (EH). Abbreviations: EN: Endosymbiont, EX: Exosymbiont, OMD: Organic matter decomposition, NC: Nutrient cycling, BAC: bacteria, Arc: Archaea, Fun: Fungi, Yea: Yeast, Fla: Flagellate, NA: Not made explicit in the paper reviewed.
